# From Maltreatment to Psychiatric Disorders in Childhood and Adolescence: The Relevance of Emotional Maltreatment

**DOI:** 10.1177/10775595221134248

**Published:** 2022-11-25

**Authors:** Franziska Schlensog-Schuster, Jan Keil, Kai Von Klitzing, Gabriela Gniewosz, Charlotte C. Schulz, Andrea Schlesier-Michel, Steffi Mayer, Stephanie Stadelmann, Mirko Döhnert, Annette M. Klein, Susan Sierau, Jody T. Manly, Margaret A. Sheridan, Lars O. White

**Affiliations:** 1 Department of Child and Adolescent Psychiatry, Psychotherapy and Psychosomatics, 70622 University of Leipzig, Leipzig, Germany; 2 Department of Educational Science, 27255 University of Innsbruck, Innsbruck, Austria; 3 Department of Neurology, Max Planck Institute for Human Cognitive and Brain Sciences, Leipzig, Germany; 4 Department of Developmental Psychology, University of Jena, Jena, Germany; 5 Department of Pediatric Surgery, 70622 University of Leipzig, Leipzig, Germany; 6 LIFE – Leipzig Research Center for Civilization Diseases, 70622 University of Leipzig, Leipzig, Germany; 7 International Psychoanalytic University Berlin, Leipzig, Germany; 8 Mt. Hope Family Center, University of Rochester, New York, NY, USA; 9 Department of Psychology and Neuroscience, University of North Carolina at Chapel Hill, Chapel Hill, NC, USA

**Keywords:** abusive parents, neglect, emotional/psychological maltreatment, internalizing, externalizing

## Abstract

Different forms of maltreatment are thought to incur a cumulative and non-specific toll on mental health. However, few large-scale studies draw on psychiatric diagnoses manifesting in early childhood and adolescence to identify sequelae of differential maltreatment exposures, and emotional maltreatment, in particular. Fine-grained multi-source dimensional maltreatment assessments and validated age-appropriate clinical interviews were conducted in a sample of *N* = 778 3 to 16-year-olds. We aimed to (a) substantiate known patterns of clinical outcomes following maltreatment and (b) analyse relative effects of emotional maltreatment, abuse (physical and sexual), and neglect (physical, supervisory, and moral-legal/educational) using structural equation modeling. Besides confirming known relationships between maltreatment exposures and psychiatric disorders, emotional maltreatment exerted particularly strong effects on internalizing disorders in older youth and externalizing disorders in younger children, accounting for variance over and above abuse and neglect exposures. Our data highlight the toxicity of pathogenic relational experiences from early childhood onwards, urging researchers and practitioners alike to prioritize future work on emotional maltreatment.

Child maltreatment ranks among the childhood adversities with the most pathogenic impact on mental health from adolescence onwards (McLaughlin et al., 2012). Yet, routine clinical practice and research predominantly focus on maltreatment exposures with more visible signs, such as physical abuse, and their mental health sequelae (Li et al., 2016). Conversely, despite their higher prevalence (Stoltenborgh et al., 2012), “hidden” subtypes, such as neglect and emotional maltreatment, have been vastly understudied (Thyen, 2008). Emotional maltreatment (EM) – also known as psychological maltreatment and mental cruelty – is difficult to define, detect and operationalize both in research and clinical practice (Baker & Brassard, 2019; Hart et al., 2017). Additionally, a tendency to minimize or underestimate EM may affect reports of caregivers and professionals as well as child protection service (CPS) records, calling for a multi-source approach in this area (Baker et al., 2021; Sierau et al., 2017). Correspondingly, despite substantial literature on physical and sexual abuse, major gaps still exist regarding the patterns of clinically relevant diagnostic outcomes of EM as well as their potential age-dependency (Cicchetti & Toth, 2016). While some recent work highlights the immediate risk for psychiatric disorders following maltreatment in early childhood (Winter et al., 2022), other work points to delayed effects (Andersen, 2016) as well as a more pervasive impact of maltreatment during adolescence (e.g., Thornberry et al., 2010). The present study seeks to address this imbalance in the literature by analyzing the relative influence of EM as compared to physical and sexual abuse as well as neglect on diagnostic outcomes among youth ranging from 3 to 16 years, with both maltreatment and clinical assessments indexed by detailed and age-appropriate reliable and valid instruments.

To date, most current research in the field has taken a cumulative risk approach suggesting that mental health outcomes are primarily determined by the quantity of risk exposure, regardless of quality or type (Evans et al., 2013). A growing body of evidence indicates that child maltreatment is a major risk factor for psychiatric illness in childhood and adolescence (Jaffee, 2017). Ample data also demonstrate that the sequelae of maltreatment may vary less as a function of differential exposures, but rather that multi- and equifinality are the rule (Vachon et al., 2015). Yet while some theoretical models acknowledge the validity of this perspective, they nevertheless emphasize the importance of subtype-specific approaches to identify differential mechanisms precipitated by disparate environmental exposures. For example, several models have specifically distinguished between deprivation, as the omission of expected interactive and cognitive input (e.g., neglect), and threat, which involves the risk or actual presence of harm to the child (e.g., abuse; Sheridan & McLaughlin, 2020). These models seek to differentiate more precisely the mechanisms by which distinct neurocognitive processes develop after threat (e.g., alterations in fear processing) versus deprivation (e.g., cognitive functioning deficits; Miller et al., 2018), ultimately leading to an increased risk for various forms of psychopathology. This argument is supported by copious research, some of which shows that threat predicts both externalizing and internalizing outcomes whereas deprivation primarily predicts externalizing outcomes (e.g., Miller et al., 2018). However, it may be surmised that the deprivation/threat distinction does not exhaustively cover all differences in childhood adversity (Sheridan & McLaughlin, 2020). Recent evidence thus supports that EM may draw on partly distinct neural networks, especially relating to social cognition (Schulz et al., 2022). We thus seek to gain purchase on the question of whether EM and its associated psychological sequelae represent an important independent dimension of early life adversities with unique explanatory value for diagnostic outcomes (Li et al., 2020; Ross et al., 2019).

EM refers to “persistent or extreme thwarting of youth’s basic emotional needs” (Barnett et al., 1993, p. 67) and can be conceptualized as a “pathogenic relational experience” (Cicchetti & Toth, 2005) in an adverse emotional climate with “the absence of the species-expected caregiver presence” (Tottenham, 2012, p. 598; White et al., 2020). EM is associated with a higher risk of depression, anxiety, attention deficit hyperactivity disorder (ADHD), posttraumatic stress disorder (PTSD) and conduct-related problems (Berzenski & Yates, 2011; Clayton et al., 2018; Gardner et al., 2019; Humphreys et al., 2020). Large-scale studies (Ross et al., 2019; Sekowski et al., 2020; Vachon et al., 2015) and meta-analyses (Infurna et al., 2016; LeMoult et al., 2020) document robust effects of emotional maltreatment on internalizing symptoms, but also on behavioural problems (Spinazzola et al., 2014). To the extent emotional maltreatment specifically interferes with developmental tasks of autonomy and identity development, some work contends that it may exert particularly strong effects during adolescence (Infurna et al., 2016; McNeil et al., 2020).

Yet, despite clear evidence and repeated bids to attend more closely to EM (Brassard et al., 2020), important gaps and methodological heterogeneity persist. Abundant research thus reports absolute rather than the relative impact of EM, i.e., without adjusting for other exposures (e.g., abuse and neglect; Infurna et al., 2016; LeMoult et al., 2020). Regarding measurement of maltreatment, the vast majority of studies involve single-source retrospective and/or questionnaire-based assessments. Likewise, regarding measurement of psychopathology, research has typically used either proximal, yet questionnaire-based symptom outcomes in youth or diagnostic, yet distal outcomes in adulthood. Also, while some studies divide EM into acts of commission and omission and/or subsume them under the respective global constructs of abuse and neglect (e.g. McNeil et al., 2020), others conceptualize EM as a unitary independent exposure without secondary subdimensions (Vachon et al., 2015). Moreover, some studies have considered information on witnessing domestic violence as part of the abuse dimension (e.g. Sheridan & McLaughlin, 2020) while others subsume it under EM, in line with well-established maltreatment coding systems (Barnett et al., 1993). Thus, despite the relatively consistent association with internalizing symptoms or disorders, these methodological inconsistencies could have potentially biased prior studies on the relationship between EM and psychopathology.

Additionally, research linking adversity to psychopathology relies predominantly on self-, parent-, and teacher-reports of psychological symptoms in keeping with a tradition of dimensional versus diagnostic approaches. Whereas dimensional approaches yield valuable insights into the impact of adversity on risk for psychopathology, psychiatric conditions are at least partly discrete phenomena and diagnoses reflect a “clinical reality”, forming the basis for assessing the clinical relevance of research findings and decision-making in clinical practice (Coghill & Sonuga-Barke, 2012; Kendler, 2018). The relative absence of research linking maltreatment to diagnosable mental illness therefore poses a major threat to the translation of maltreatment-related research to clinical practice.

## The Current Study

The present study seeks to address the aforementioned gaps on a sizable sample of 3–16 - years-olds. To this end, we combine fine-grained, multi-source maltreatment assessments in childhood or adolescence with valid and reliable diagnostic assessments manifested in youth as young as preschool and early school-age. In so doing, our study aims to address the following key research questions using a latent variable approach:

(1) Does the more strongly relational dimension of EM account for additional variance in diagnostic outcomes (especially in regard to internalizing disorders) over and above abuse (threat) and neglect (deprivation)? If yes, is this additional variance best conceptualized as an independent construct or subsumed under threat and deprivation by distinguishing between emotional abuse and neglect?
(2) Do psychiatric diagnoses following child maltreatment manifest immediately in development or primarily after a delay in adolescence as some previous research implies (see Andersen, 2016)?

To facilitate comparison with other studies, we first sought to delineate how the risk for various psychiatric disorders increases as a function of overall (global) maltreatment, as well as of individual maltreatment exposures (i.e., abuse, neglect and EM). Second, using a step-by-step approach, we aimed to disentangle the relative effects of abuse, neglect and EM on psychiatric disorders. In line with previous work, we expected that before taking EM into account, abuse and neglect would independently predict the risk of internalizing and externalizing disorders, with abuse exerting a stronger effect than neglect on internalizing disorders (e.g. Miller et al., 2018). Next, we sought to test whether this pattern was robust to partitioning EM into acts of commission and omission, subsuming these under abuse and neglect factors, respectively. After this, we modelled EM as a distinct dimension, predicting it would explain overlapping as well as additional variance in internalizing and externalizing disorders, over and above the effects of abuse and neglect. Moreover, in light of recent suggestions that EM may prove particularly detrimental during adolescence (e.g., McNeil et al., 2020), we also explored possible age-related patterns.

## Method

### Participants

The sample comprised *N =* 778 children and adolescents (*M*_age_ = 8.91, *SD*_age_ = 3.09, 47.3% females) as well as their primary caregivers, taking part in the project *Analyzing Pathways from Childhood Maltreatment to Internalizing Symptoms and Disorders in Children and Adolescents* (AMIS; White et al., 2015) which seeks to analyze developmental pathways from childhood maltreatment to psychiatric symptoms and disorders (see Table 1 and Figure S1 for sample and maltreatment characteristics). The sample included *n =* 306 (39.3%) children and adolescents with and *n =* 472 (60.7%) without known maltreatment histories, recruited via child protection services (CPS; *n* = 162, 90.7% maltreated), child and adolescent psychiatric services (CAPS; *n* = 121; 50.4% maltreated), and the community, from daycare centers, general practitioners, and the resident registration office (*n* = 495; 19.8% maltreated) of Leipzig and Munich. Leipzig is an eastern German city with a population of approximately 500.000 inhabitants and above-average rates of poverty. Munich is a metropolis with a population of 1.35 million in southern Germany with lower poverty rates but higher proportions of immigrant families than Leipzig. The CAPS subsample comprised school-age children and adolescents. Extending the psychiatric risk group to preschool and early school age, a subset of community children (*n* = 86; 23.3% maltreated) were oversampled for internalizing symptoms above the borderline cut-off on the Strengths and Difficulties Questionnaire (Goodman, 1997; Klitzing et al., 2014). Supplementary Figure S1 illustrates the prevalence of EM (*n* = 248, 81%), neglect (*n* = 196, 64%) and abuse (*n* = 129, 42.2%) as well as the degree of overlap between these subtypes (*n* = 73, 24% EM+NEG; *n* = 31, 10% EM+ABU; *n* = 80, 26% EM+ABU+NEG). EM shows the highest degree of single-subtype maltreatment (*n* = 64, 21%) compared to neglect (*n* = 40, 13%) and abuse (*n* = 15. 5%).

**Table 1.** Demographic and psychiatric disorder and maltreatment subtypes of the total sample (*N* = 778), total maltreated (*n* = 306), total non-maltreated (*n* = 472), and non-maltreated youth without high-risk due to oversampling and recruitment in youth psychiatric services (*n* = 335).

Sample size	Total sample	Maltreated	Non-maltreated	Non-maltreated w/o hi risk
	*N* = 778	*n* = 306	*n* = 472	*n* = 335
Demographics				
Child age (mean,SD)	8.91 (3.09)	9.45(3.08)	8.56 (3.06)	8.67 (2.95)
Child gender female (n, %)	368 (47.3)	130 (42.5)	238 (50.4)	172 (51.3)
School education caregiver (median)	Upper secondary	Upper secondary	High school diploma	High school diploma
Psychopathology (n, %)				
Externalizing diagnosis	152 (19.5)	92 (30.1)	60 (12.7)	22 (6.6)
Internalizing diagnosis	252 (32.4)	124 (40.5)	128 (27.1)	49 (14.6)
Maltreatment subtypes (n, %)				
Abuse	129 (16.6)	129 (42.2)	N/A	N/A
Physical abuse	107 (13.8)	107 (35.0)	N/A	N/A
Sexual abuse	41 (5.3)	41 (13.4)	N/A	N/A
Neglect	196 (25.2)	196 (64.1)	N/A	N/A
Failure to provide	100 (12.9)	100 (32.7)	N/A	N/A
Lack of supervision	147 (18.9)	147 (48.0)	N/A	N/A
Moral/legal/educational	31 (4.0)	31 (10.4)	N/A	N/A
Emotional maltreatment	248 (31.9)	248 (81.0)	N/A	N/A

### Procedure

Families attended two assessments, which lasted about 3 hours and comprised interviews, questionnaires, and experimental tasks. Psychiatric and maltreatment interviews were not collected as part of the same assessment. For the CAPS and the community sample, the psychiatric assessments were conducted first (Wave 1), followed by the maltreatment interview (Wave 2) spaced on average 1 year apart (M = 1.18 years, *SD* = .862). For families recruited via the CPS, maltreatment interviews were conducted as part of the same wave (but on a separate day) as the psychiatric interview (see Table S1 for detailed information). Using the detailed information on timing of maltreatment (see below), we removed any maltreatment incidents that occurred later in development than the diagnostic interview, to safeguard against reversal of temporal order (i.e., clinical disorder preceding maltreatment). The maltreatment interviews were coded after the appointment. The institutional review board (IRB) of the university provided ethical approval for the study. Prior to participation, informed oral and written consent was obtained from caregivers and youth. Caregivers were reimbursed, and youth received a gift as compensation for their time.

### Measures

*Maltreatment classification:* Maltreatment was operationalized using the *Maltreatment Classification System* (MCS; Barnett et al., 1993), a valid and reliable coding manual providing semantic definitions and exemplars of six subtypes (Failure to Provide, Lack of Supervision, Sexual Abuse, Physical Abuse, Emotional Maltreatment (EM), Moral-legal/Educational Maltreatment; see Table S2 for definitions and examples; Manly et al., 2013). To ensure high quality and international comparability of ratings, the AMIS group was trained and continually supervised by an author of the MCS (Jody T. Manly, PhD) throughout the AMIS project. Reports of potential maltreatment incidents were ascertained from two sources: First, for CPS-referred youth (*n* = 162), official CPS files were retrieved and analyzed using the MCS. Second, for all youth (including those with CPS referral), the *Maternal Maltreatment Classification Interview* (MMCI; Cicchetti et al., 2003) was conducted with caregivers by trained Masters-level research assistants and videotaped for subsequent coding. Information gathered from both sources was used to derive chronicity, timing and severity, of maltreatment incidences, individually for each subtype defined in the MCS.

The MMCI comprises 10 standardized screeners with follow-up questions in the case of positive response to assess the lifetime presence of maltreatment incidents. For each incident, coders rated the subtype, severity (1 = low to 5 = high), and developmental period in which the incident occurred, i.e., infancy (0–1.4 years), toddlerhood (1.5–2 years), preschool age (3–5 years), early school age (6–7 years), late school age (8–12 years), and adolescence (13–18 years). In addition to maximum severity and number of subtypes, an index of chronicity per subtype was derived (i.e., the proportion of periods affected by a subtype relative to the total number of experienced periods). Notably, the MCS explicitly aims to minimize confounding between subtypes by requiring identification of a distinct act of the caregiver to substantiate an individual subtype. Although all forms of maltreatment may involve an emotional aspect, the MCS stipulates that coding an act as EM excludes other subtypes, forcing coders to reach a decision on whether the act in question is best classified as EM or a different subtype. Moreover, we appended a novel distinction between emotional abuse (e.g., extreme rejection/devaluation of the child) and emotional neglect (e.g., extreme disregard for emotional needs of the child) to the MCS, which was employed solely for analyses regarding Model 3 (see Table S2 for detailed information on definitions and examples of subtypes).

If coding problems occurred, a group of senior researchers chaired by the deputy principal investigator of the study discussed the case and, if necessary, consulted Dr. Manly. Independent blind raters double-coded 20% of the interviews and records, which yielded a moderate to high inter-rater agreement for maltreatment (CPS file codings: Cohen’s *k* between .58 and .78; caregiver interviews Cohen’s k between .78 and 1.00; Sierau et al., 2017). For CPS-referred youth, all information available from case records and interviews was pooled at the incident level, using the source that provided more information on a subtype and its severity for each developmental period. If the CPS record indicated maltreatment, but none was reported on the MMCI, maltreatment ratings were based solely on CPS record (*n* = 6). As the MCS subsumes witnessing domestic violence (WDV) under EM, but other research (Sheridan & McLaughlin, 2020) subsumed WDV under abuse, we also coded the presence of WDV to additionally derive an EM variable that excluded all incidents related to WDV, as indexed by the MMCI.

*Psychiatric diagnoses:* To rate psychiatric diagnoses, trained Masters-level research assistants conducted semi-structured clinical interviews with caregivers about the potential symptoms and disorders of their children. Symptoms and diagnoses of the children were coded as present/absent based on the algorithms detailed in the manuals. For younger youth (3–8 years) we conducted the Preschool Age Psychiatric Assessment (PAPA; Egger & Angold, 2004) to derive diagnoses made over the last 3-month primary period. For school-aged youth (8–16 years) we used the *Schedule for Affective Disorders and Schizophrenia* for school-age youth (Kiddie-SADS-Present and Lifetime Version; K-SADS-PL; Kaufman et al., 1997). For the present study, we used K-SADS-PL data only for current diagnoses. Both interviews are based on DSM-IV diagnostic criteria (American Psychiatric Association, 2000) and comprise screening questions on key symptoms of each disorder followed up by diagnostic supplements in the case of positive screenings.

### Data Analysis

All analyses were carried out using *R* (version 3.6.1.; R core Team, 2019). To clinically characterize our sample, we preliminarily computed odds ratios (OR) for the propensity of clinical diagnoses (only diagnostic categories with at least *n* = 30 participants included) following overall maltreatment, abuse, neglect and EM across the complete sample using the *DescTools* package (Signorell et al., 2021). Additionally, we computed ORs for the propensity of clinical diagnosis (only diagnostic categories with at least *n* = 20 participants included) following maltreatment for younger (3–8 years) and older children (9–16 years).

For all subsequent steps, we conducted structural equation modeling (SEM) using the *lavaan* package (Rosseel, 2012). We modeled several latent factors reflecting internalizing (INT) and externalizing disorders (EXT) as well as maltreatment (MAL; including information on emotional maltreatment), abuse and neglect excluding information on emotional maltreatment (ABU, NEG). Additionally, we specified two alternative latent factors reflecting abuse and neglect factors (ABU+, NEG+) that included information on emotional abuse and emotional neglect as well as one unitary factor reflecting emotional maltreatment (i.e., including information on emotional abuse and neglect; EM). We specified INT based on three categorical indicators reflecting the presence of (a) depressive, (b) anxiety, and (c) other disorders related to the internalizing spectrum (e.g., bipolar, eating, inhibited reactive attachment disorder). Likewise, EXT was specified using three categorical indicators reflecting presence of (a) conduct disorder, (b) oppositional defiant disorder, and (c) other disorders related to the externalizing spectrum (e.g., attention-deficit/hyperactivity, substance use, disinhibited reactive attachment disorder). Maltreatment dimensions were specified using three indicators: number/presence of subtypes, maximum severity, and chronicity (for additional information on structural equation modelling see Supporting Information).

SEM analyses were carried out in two steps. First, to confirm the well-established association between general and subtype-specific maltreatment experience and psychiatric diagnosis in prior studies, we regressed INT and EXT on MAL (Model 1), ABU and NEG (Model 2) as well as ABU+ and NEG+ (Model 3) and EM (Model 4). Second, to analyze the relative predictive value of emotional maltreatment on diagnostic outcomes, we compared path coefficients of abuse and neglect (i.e., ABU, NEG, ABU+, NEG+) with diagnostic outcomes (i.e., INT and EXT) between models (i.e., Model 2, 3 and 4). We also compared Model 4 to an alternative model with unstandardized path coefficients fixed to zero (Model 4a) using an adjusted χ^*2*^-difference test (Satorra & Bentler, 2010) that has been suggested for ordinal outcomes (Pavlov et al., 2020). Finally, in two supplementary analyses, we reanalyzed Model 4 while excluding all incidents related to or accompanied by WDV from EM (i.e., Model 4b) as well as using a multi-group analysis to compare effects among 3 to 8-year-olds versus 9 to 16-year-olds (i.e., Model 4c).

For all but the first model, auto-correlated residuals (Sörbom, 1975) were specified among the corresponding observed indicators of maltreatment dimensions. Additionally, all models were adjusted for age at diagnostic interview, gender, and caregiver education. Due to the categorical and ordinal nature of some of the latent variables, we employed a weighted least squares estimator (WLSMV) across all SEMs. All models were evaluated using the chi-square statistic, comparative fit index (CFI), root-mean-squared error of approximation (RMSEA), and the standardized root-mean-squared residual (SRMR). According to, Hu and Bentler (1999) a RMSEA ≤ 0.05 (0.08), a CFI ≥ 0.95 (0.90), and a SRMR ≤ 0.05 (0.08) represent a good (adequate) model fit.

## Results

### Preliminary Analysis

Supplementary Figures S2–S6 summarize the results of the preliminary analyses, showing that maltreatment as well as each of the three maltreatment subtypes (abuse, neglect, EM) coincided with elevated odds of developing psychiatric disorders from both the internalizing (except in the case of abuse) and externalizing spectrum. Furthermore, the age-specific ORs indicate that maltreatment is associated with elevated odds of developing psychiatric disorders from both the internalizing and externalizing spectrum for younger as well as older children.

### Main Structural Equation Modeling Analyses

All models showed good or adequate fit to the data (see Table S3). Model 1 focused on global maltreatment (i.e., without subtype distinctions), indicating that maltreatment exerted an effect on both internalizing (β = .19, *p =* .009) and externalizing disorders (β *=* .36*, p ≤* .001). Additionally, younger age was associated with internalizing disorders (β = −.15, *p* = .009) while male gender was associated with externalizing disorders (β = −.27, *p* ≤ .001; see Table S3, Figure S7).

**Figure 1.**
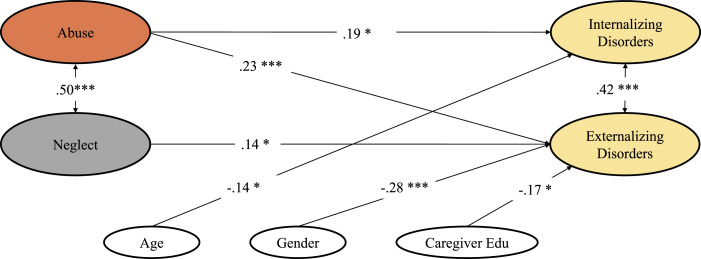
Structural equation model 2 predicting psychiatric outcomes from abuse (excluding emotional abuse) and neglect (excluding emotional neglect) experiences after controlling for age of diagnosis, gender, and caregiver education.

Model 2 distinguished between abuse and neglect without considering information on emotional maltreatment. Results demonstrate that internalizing disorders were predicted by abuse only (β = .19, *p =* .012), while externalizing disorders were predicted by both abuse (β = .23, *p* ≤ .001) and neglect (β = .14, *p =* .020), with abuse exerting a stronger effect. Comparable associations emerged for covariates, as in Model 1. Additionally, lower caregiver education was associated with externalizing disorders (β = −.17, *p =* .011; see Table S3, Figure 1).

Model 3 again distinguished between abuse and neglect with emotional abuse and neglect subsumed under the corresponding dimensions. Results demonstrate that internalizing and externalizing disorders were predicted by abuse only, with abuse exerting a stronger effect on internalizing disorders (β = .30, *p* ≤ .001) and externalizing disorders (β = .28, *p* ≤ .001) than in Model 2. The effect of neglect on externalizing disorders was abolished in comparison to Model 2. Comparable associations emerged for covariates as in Model 2 (see Table S3, Figure S8).

**Figure 2.**
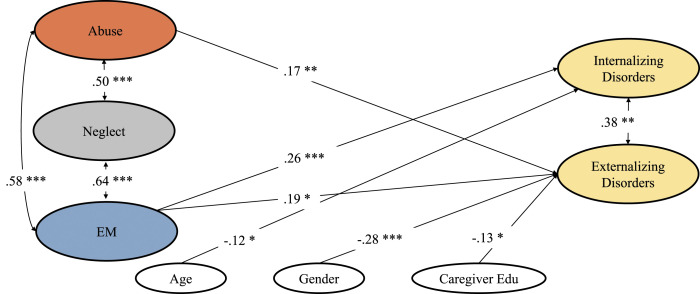
Structural equation model 4 predicting psychiatric outcomes from abuse, neglect and emotional maltreatment experiences after ontrolling for age of diagnosis, gender, and caregiver education.

Model 4 distinguished between abuse, neglect, and EM, indicating that EM was the only subtype predicting internalizing disorders (β = .26, *p* = .008), while EM (β = .19, *p* = .023) and abuse (β = .17, *p* = .006) were associated with externalizing disorders. Notably, as compared to Model 2 and 3, the relationship between abuse and internalizing disorders (Model 2 and 3) as well as neglect and externalizing disorders (Model 2) was abolished after accounting for EM. Comparable associations emerged for covariates as in Model 2 and 3 (see Table S3, Figure 2). Additionally, results demonstrate that the less restrictive model with (freely estimated) effects of EM on diagnostic outcomes (Model 4) better represents our data than the more restrictive alternative assuming no association between EM and diagnostic outcomes (Model 4: χ^2^(101) = 142.49; Model 4a: χ^2^(103) = 162.23; *p* = .002). Also, altering EM to exclude incidents related to witnessing domestic violence resulted in a model in which EM continued to predict internalizing disorders while the path to externalizing disorders was abolished (see Table S4). Finally, a multi-group analysis partitioning our sample into younger (3 to 8-year-olds) and older age groups (9–16-year-olds) revealed that the effect of EM on internalizing disorders surfaced more clearly in older (β = .29, *p* = .005) than younger participants (β = .19, *p* = .100). Additionally, EM was associated with externalizing disorders among younger (β = .27, *p* = .021), but not older participants (β = .09, *p* = .347). Conversely, the effect of abuse on internalizing disorders was merely present among younger participants (3 to 8-year-olds: β = .16, *p* = .020; 9 to 16-year-olds: β = .014, *p* = .895), whereas its effect on externalizing disorders was merely present among older participants (3 to 8-year-olds: β = .03, *p* = .765; 9 to 16-year-olds: β = .30, *p* ≤ .001; see Table S5).

## Discussion

Ours is the first large-scale study in a sizable sample of youth ranging from 3 to 16 years to examine standardized diagnostic outcomes of abuse, neglect, and emotional maltreatment (EM) manifested in early childhood and adolescence. To this end, we used fine-grained multi-source dimensional maltreatment assessments and validated age-appropriate clinical interviews to diagnose psychiatric disorders. Besides confirming known patterns regarding effects of maltreatment on psychopathology, our results underscore EM as a prominent risk factor with the strongest association with internalizing and externalizing disorders over and above physical forms of abuse and neglect.

Our findings suggest that overall as well as exposure-specific maltreatment experiences predict internalizing and externalizing disorders which converges with a vast range of studies (e.g., Struck et al., 2020). Nevertheless, this study extends the existing research in at least two ways: First, to the best of our knowledge, only few studies to date have used structural equation modelling (SEM) and a latent variable approach on a combination of fine-grained maltreatment and clinical interview data in samples ranging from early childhood to adolescence (Rivera et al., 2018). Inasmuch as SEM accounts for measurement error, affords estimation of latent variables (e.g., maltreatment) from observed indices (e.g., chronicity), and allows for evaluation of model fit, this underscores the likely robustness of our effects. As such, our results demonstrate that the risk for clinical disorders following maltreatment can already unfold in early and middle childhood, highlighting the need for early intervention (Moss et al., 2011). Second, our step-by-step data-analytic approach allowed us to disaggregate the relative effects of abuse, neglect, and EM. More specifically, at the level of clinical disorders, our data offer implications as to whether EM is best subsumed under abuse and neglect or provides a dimension in its own right.

Regarding our exposure-specific findings, our data confirm the association of abuse with internalizing disorders while externalizing disorders were related to abuse and neglect (Model 2), which is in line with other studies adopting a DMAP perspective (Milojevich et al., 2019; Sheridan & McLaughlin, 2020). Conversely, neglect did not exert an effect on internalizing disorders, which is in line with some, but not other previous studies (Miller et al., 2018, 2020; Vachon et al., 2015). Possibly, the exposure-specific pattern documented here and by Miller et al. (2018) maps onto underlying mechanisms, whereby neglect is thought to primarily exert its effects via cortically mediated functions (e.g., executive functioning) whereas effects of abuse also hinge on subcortical functions (e.g., fear learning; Sheridan & McLaughlin, 2020). Potentially, discrepancies with findings showing non-specific effects may be attributable to sampling factors (see Barnett et al., 1993) with some studies focusing exclusively on documented instances of child maltreatment (e.g., Miller et al., 2020; Vachon et al., 2015), that typically over-represent severe cases with high subtype co-occurrence. By contrast, in line with Miller and colleagues (2018), our study also included many cases from the community with low to medium severity and fewer subtypes, potentially positioning this work to better disentangle exposure-specific patterns.

Remarkably, the comparison between Model 2 and Model 3 suggests that differences in the conceptualisation and/or definition of abuse and neglect may have a substantial impact on results. In our data, the association of abuse with clinical outcomes, especially internalizing disorders rose markedly when information on emotional abuse were taken into account. In contrast, the effect of neglect was abolished after taking information on emotional neglect into consideration (potentially also due to the predictive power of the abuse construct after subsuming emotional abuse under it). It is difficult to assess whether these findings are specific to our study or if other data would yield comparable results.

Crucially, our study expanded on previous research by a detailed analysis of the relative effects of EM, abuse and neglect. With an eye to the odds ratios (ORs; Figures S2–S5), we first note that abuse showed the strongest effect on externalizing disorders whereas all three types of exposures (abuse, neglect, and EM) exerted similar effects on internalizing disorders. Yet, while these ORs may primarily prove clinically informative – inasmuch as they reflect what diagnostic patterns were observed following specific exposures – they did not invariably correspond with our multivariate analyses. Thus, jointly modeling different maltreatment exposures as dimensions and co-varying for sociodemographics (Model 4, Figure 2) yielded a pattern whereby the effect of EM on internalizing disorders now clearly exceeded those of abuse and neglect. Indeed, for internalizing disorders, we found that EM not only exerted the strongest influence relative to other subtypes, but also explained additional variance over and above abuse and neglect (Model 2 vs. Model 4). This dovetails with large-scale studies in adults (Keyes et al., 2012) and meta-analyses showing specific effects of EM on depression (e.g., Infurna et al., 2016; LeMoult et al., 2020; Sekowski et al., 2020). Our study therefore extends these patterns downwards to early childhood, supporting the prognostic importance of EM.

Turning to our age-related analyses, we first note that the odds ratios (ORs; Figure S6) suggested that maltreatment coincides with increased rates of psychiatric disorders, with ORs for internalizing disorders ostensibly somewhat higher in younger than in older participants. To reiterate, these ORs may prove clinically informative and highlight early diagnostic sequelae of maltreatment (Winter et al., 2022). They do not, however, mesh well with the age-related increases in internalizing disorders widely documented in the literature (e.g., Hankin et al., 2015; Thapar et al., 2012).

Indeed, when pitting our age-specific ORs (Figure S6) against the age-related multi-group analyses (Table S5) we note three important differences: First, our age-related multi-group analysis only partly aligns with a stronger maltreatment-related effect on internalizing disorders for younger versus older participants. Thus, the overall effect of EM on internalizing disorders was more clearly attributable to the older than the younger age group. This potentially concords with a lifespan perspective, whereby internalizing disorders become more susceptible to the influence of EM during late childhood/adolescence, potentially interfering with developmental tasks of autonomy and identity-formation as well as peer competence (e.g., McNeil et al., 2020).

Second, the aforementioned pattern notwithstanding, our results also emphasize the relevance of EM for immediate diagnostic outcomes during early childhood. Thus, besides making a trend-level contribution to the overall EM effect on early internalizing disorders, EM was also associated with externalizing disorders among younger, but not older participants. Initially, EM may thus act more broadly, for example, conferring risk to “childhood-limited” antisocial behavior, in turn, predisposing to developmental cascades involving low peer competence and later internalizing disorders (see Moffitt, 2006).

Third, abuse was merely associated with internalizing disorders among younger participants, whereas its association with externalizing disorders was merely present among older participants. To the best of our knowledge, no such pattern has previously been reported. Moreover, this pattern may also come as a surprise, in light of the well-established links between adverse parenting and early-onset life-course persistent antisociality (see Moffitt, 2006). However, we note that approximately half of our older age group was still in late childhood (aged 9–12), suggesting that most externalizing disorders within this age-group could still be considered “childhood-onset”. Nevertheless, we speculate that this pattern may potentially reflect an initial effect of physical abuse on internalizing disorders followed by a delayed effect on externalizing disorders (Andersen, 2016). This pattern may correspond to views that early submissiveness and over-compliance may minimize risk as long as children still lack the resources to evade or engage with (intra-familial) threats (Badanes et al., 2011; Keil et al., 2019; White et al., 2020).

More broadly, our findings provide additional evidence at the level of clinical disorders, suggesting that it might prove worthwhile to consider EM as an independent dimension that complements threat and deprivation, especially for internalizing disorders. Indeed, even after removing a potential threat component of EM (i.e., witnessing domestic violence; WDV), the path from EM to internalizing disorders remained intact. Our results thus corroborate the notion that one core aspect of maltreatment involves a pathogenic relational experience (Cicchetti & Toth, 2005) which may potentially interfere with the caregiver functioning as a supportive source of co-regulation with a variety of detrimental sequelae (e.g., attributional biases, reduced self-worth), particularly relevant to internalizing disorders (White et al., 2020).

Conceptually, a detrimental impact of EM is readily conceivable for relational, representational, mentalizing, and self-related processes, potentially originating from the internalization of a devaluing or unsupportive caregiver (e.g., Kim & Cicchetti, 2006; Riggs, 2010; Wright et al., 2009). These negative appraisals of the self may manifest over time via *internal working models* (Bowlby, 1973) and are thought to relate to elevated feelings of shame (Gilbert, 2005), self-blame (Benjamin, 2003) or pathogenic guilt (Zahn-Waxler & Van Hulle, 2011), ultimately predisposing to depression, among others (Ross et al., 2019). Intriguingly, this interpretation is not only supported by various studies using self-reports and interviews (e.g., Ross et al., 2019; Sekowski et al., 2020) but also meshes with a behavioral hyper-cooperative or self-sacrificing strategy detected using an objective game-theoretical task among primarily emotionally maltreated children (Keil et al., 2019). Moreover, recent neuroimaging data support a specific influence of EM (even after controlling for abuse and neglect) on elevated neural activation in regions linked to aversive affects and internally-oriented processes (e.g., mental state attribution) during a social stressor (Schulz et al., 2022). Thus, these processes may underpin developmental trajectories, especially with internalizing outcomes and do not fully overlap with those proposed in the deprivation/threat distinction (Sheridan & McLaughlin, 2020).

Although not at the heart of our analysis, our supplementary analysis which removed the effect of WDV from EM additionally raises further methodological questions. While our results showed that the association of EM with internalizing disorders remained intact, the effect decreased so that abuse elicited the strongest associations with both, internalizing and externalizing disorders. The effect of EM thus seems to be partially driven by incidents of WDV, especially for externalizing disorders. This seems noteworthy as the MCS conceptualises WDV as part of EM while other studies have considered it as part of emotional abuse (Sheridan & McLaughlin, 2020). However, together with the aforementioned neuroimaging results which also reported similar effects of EM independent of WDV, this indicates that at least some of the pathogenic effects of this type of exposure are attributable to less “debatable” kinds of EM. Interestingly, our pattern of findings appears to be difficult to reconcile with a pure cumulative risk perspective on maltreatment, given that EM had the highest degree of single-subtype maltreatment exposures in our sample (see Figure S1).

Some key limitations deserve attention. First, data were collected cross-sectionally indicating that causal claims should be made with caution. However, our maltreatment data partly builds on prospective longitudinal data as events in CPS records were collected before the onset of the study. Similarly, although caregiver interviews were retrospective, the highest rates of reported maltreatment occurred in early childhood. Given that the vast majority of our youth were older at outcome assessment, longitudinal effects seem likely.

Second, our sample composition was not representative of the population, spanning youth from the community as well as at-risk backgrounds. Importantly, however, our study aimed to estimate the sequelae of maltreatment (subtypes) for psychiatric disorders. Our recruitment strategy served to saturate our sample with sufficient psychiatric and maltreatment-related risk targeting highly burdened families who rarely participate in research. This may be especially true for our high risk families recruited via CPS, which often face many psychosocial problems. Accordingly, our findings may arguably be characterized by enhanced clinical relevance, shedding light across the full spectrum of maltreatment. With regard to our age-related patterns, we also note that approximately half of our older age group was aged 9–12 years which may account for discrepancies with the adolescent literature on maltreatment. This over-representation of pre- and early adolescence alongside the upper age-limit of 16 years in our sample may have placed an upper-bound on detecting the adolescent-typical rise in internalizing, specifically depressive disorders, often reported in the literature. Thus, for example, in the Dunedin longitudinal study the most pronounced rise for depression occurred at age 16–18, but not before (Thapar et al., 2012), a pattern also documented in other longitudinal studies (e.g., Hankin et al., 2015). In any case, we feel caution is warranted when interpreting our age-related analyses, given their post-hoc nature and the cross-sectional nature of our data which may have given rise to potential cohort and method effects (e.g., use of the PAPA vs. the K-SADS-PL, in younger vs. older participants, respectively).

Third, as the caregiver served as one key informant for maltreatment and the sole informant for psychopathology, our results may also be affected by a shared method bias, which could have unduly inflated associations between our constructs of interest. That said, both our maltreatment and diagnostic interviews were semi-structured and interviewer-based, whereby the coder determines the presence/absence of a phenomenon. While far from perfect, utilization of interviewer-based assessments may provide a partial solution to the dilemma between the indispensability of the caregiver perspective (especially among young children with limited verbal competencies) and the biases introduced by an over-reliance on caregivers as informants (given their potentially dual role as perpetrators).^1^

Fourth, our operationalization of measuring EM may reduce relational experiences to their most negative aspects as they were recalled by caregivers and reported in CPS records and therefore should not be equated with more global measures of (internal representations of) caregiving relationships (e.g., attachment). However, given its availability in large-scale samples, EM may offer some of the strongest support for the hypothesis that the pathogenic core of maltreatment may be best characterized as a toxic relational experience. Likewise, emotional or psychological maltreatment have proven particularly difficult to define with ample debates in the field, such as on the distinction between “poor parenting” as compared to EM (Cicchetti, 1991). However, the rigorous MCS approach used in this study stipulates that coding an incident as EM excludes other maltreatment subtypes. In turn, this prevents inflationary identification of EM which could otherwise be said to accompany any form of maltreatment (Barnett et al., 1993). It is striking that EM remained the most predictive factor for internalizing disorders despite this conservative coding approach. At the same time, disaggregating EM into separate latent factors involving omission (emotional neglect) and commission (emotional abuse) was not possible, primarily owing insufficient cell sizes, highlighting the limitations of such a conservative approach. Future work in larger samples may wish to add a higher resolution to EM by distinguishing between emotional abuse and neglect, but an important caveat to keep in mind is that such attempts will place an even greater burden on achieving definitional consensus, not only regarding EM, but also its possible subdimensions.

## Conclusion

In conclusion, emotional maltreatment (EM) still represents an underappreciated subtype that may interfere with the species-expected need for a caregiving relationship. Acting most strongly on internalizing disorders, our data suggest a central role for EM. The high level of early dependence on the parent-child relationship may therefore place EM at the heart of the pathogenic influence of maltreatment experiences, alerting practitioners to the importance of taking it into account for decision-making in clinical practice. However, despite significant advances in our knowledge regarding the developmental sequelae and intervention following (emotional) maltreatment, translation to social, clinical, and legal work practices still lags behind this knowledge (Baker et al., 2021). One reason for this is that there remains no internationally accepted definition of emotional/psychological maltreatment that has been stringently incorporated into state laws while respecting cultural differences (Baker & Brassard, 2019). Such an international consensus, alongside appropriate training and education of professionals regarding definition and measurement of this maltreatment subtype is indispensable to optimizing detection, access to, and implementation of intervention for affected children (Manly et al., 2021). Second, primary and secondary prevention and intervention programs must prioritize targeting the quality of the parent-child relationship over the child in isolation as a source of child well-being and the child’s psychological, emotional, and physiological health. Indeed, as outlined in detail elsewhere (Brassard et al., 2020; Valentino, 2017), a tiered model of service delivery whereby efficacious brief relational interventions (e.g., Reminiscing and Emotion Training; RET; Valentino et al., 2019) may be offered as a first line of treatment and supplemented by more intensive, long-term intervention (e.g., Child Parent Psychotherapy; Lieberman et al., 2015) only when families do not respond, holds particular promise.

## Supplemental Material

Supplemental Material - From Maltreatment to Psychiatric Disorders in Childhood and Adolescence: The Relevance of Emotional Maltreatment Supplemental Material for From Maltreatment to Psychiatric Disorders in Childhood and Adolescence: The Relevance of Emotional Maltreatment by Franziska Schlensog-Schuster, Jan Keil, Kai Von Klitzing, Gabriela Gniewosz, Charlotte C. Schulz, Andrea Schlesier-Michel, Steffi Mayer, Stephanie Stadelmann, Mirko Döhnert, Annette M. Klein, Susan Sierau, Jody T. Manly, Margaret A. Sheridan, and Lars O. White in Child Maltreatment

## Note

1 In a follow-up analysis (available from the corresponding authors), we limited ourselves to maltreatment-related information derived from CPS files, hence ruling out a shared method bias. This analysis continued to show an incremental predictive effect of EM. However, the effect now interestingly emerged for externalizing rather than internalizing diagnoses. We tentatively conclude that the caregiver perspective on maltreatment – besides allowing for broader sampling of the full spectrum maltreatment beyond CPS detected cases – may add additional information on EM that is crucial for the prediction of internalizing diagnoses. In a speculative vein, this may be partly due CPS files favoring the detection of certain more readily observable forms of EM (e.g., witnessing domestic violence) over others that are less readily observable (e.g., denigrating/ devaluing the child, role-reversal, undermining autonomy) while MICM interviews offer a broader perspective on EM, in particular.
