# Supplementary material for: From Maltreatment to Psychiatric Disorders in Childhood and Adolescence: The Relevance of Emotional Maltreatment
Source: Child Maltreat. 2022 Nov 25;29(1):142–54. doi: 10.1177/10775595221134248 (PMC10895962; doi:10.1177/10775595221134248)
Supplement: Supplemental Material - From Maltreatment to Psychiatric Disorders in Childhood and Adolescence: The Relevance of Emotional Maltreatment [file sj-pdf-1-cmx-10.1177_10775595221134248.pdf]

Supplement

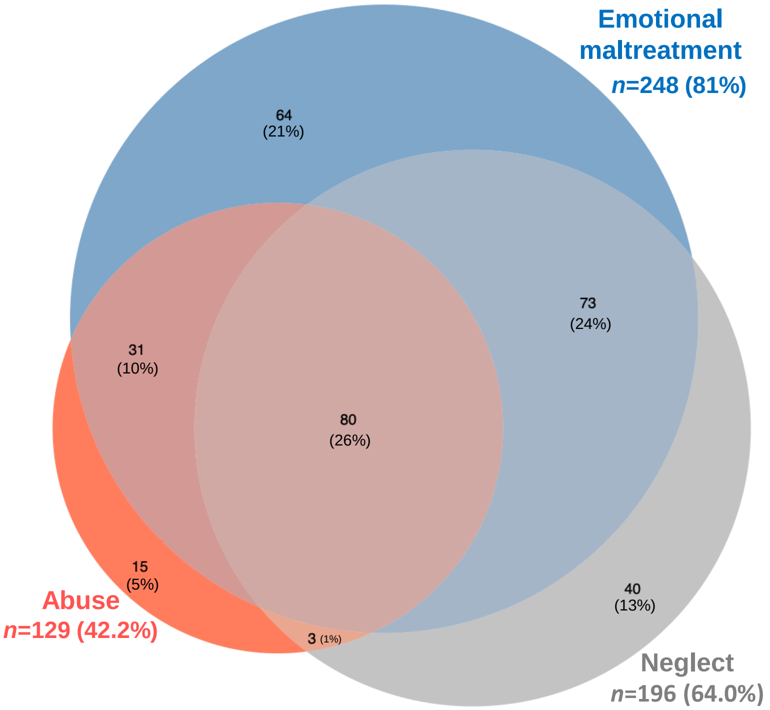

Figure S1. Venn diagram illustrating the degree of overlap between emotional maltreatment (EM), abuse, and neglect in the sample with caseload for subtypes displayed. Please note: Presence/ absence of maltreatment subtype used for this figure, but continuous scales (chronicity, severity, subtype number) used for analyses.

Table S1. Illustration of recruitment strategy across time points of the [redacted]-study [redacted].

**Wave 2009 - 2012 (N=1076)**	**Wave 2012 - 2015 (N=869)**
**Subsample 1 (low risk; n = 325)**<br>• Recruitment: Community (oversampled for internalizing symptoms)<br>• Age: 3-6 years<br>• Psychiatric Assessment: PAPA	Reassessment Subsample 1 (n = 236)<br>• Age: 4-8 years<br>• Maltreatment assessment: Interview via MCS
**Subsample 2 (medium risk; n = 751)**<br>• Recruitment: psychiatric refferals & community<br>• Age: 8-14 years<br>• Psychiatric Assessment: Kiddie-SADS	Reassessment Subsample 2 (n = 471)<br>• Age: 9-16 years<br>• Maltreatment assessment: Interview via MCS
	**Subsample 3 (high risk; n = 162)**<br>• Recruitment: via Child Protection Services<br>• Age: 4-16 years<br>• Psychiatric assessment: PAPA (4-8 years), Kiddie-SADS (9-16 years)<br>• Maltreatment assessment: Interview via MCS

Table S2. Summary of dimensions, subtypes, definitions and examples of maltreatment as indexed by the Maltreatment Classification System (Barnett et al., 1993).

**Dimensions**	**Subtypes**	**Definitions**	**Examples (Severity)**
**Abuse**	Sexual abuse	(Attempted) Sexual contact between child and caregiver for the adult's sexual gratification or financial benefit	Sexual intercourse with the child (4)
	Physical abuse	Responsible adult inflicts physical injury on child by non-accidental means (i.e., negligence or accident).	Caregiver hits child with a belt leaving bruises on the child's backside (2)
**Neglect**	Failure to provide	Neglect of the child's physical needs in terms of suitable and adequate food, clothing, housing, medical care, proper and adequate hygiene.	Child does not receive lunch or dinner 2-3 times per week (2)
	Lack of supervision	Inadequate supervision of a child by an inappropriate unsuitable caregiver (is alcoholic; has already committed violent acts against children).	Elementary school child left alone overnight (4)
	Moral-Legal Education Maltreatment	Caregiver exposes child to illegal/criminal activities that promote or involve criminal or antisocial behavior in child. Lack of guarantee of age-appropriate training.	Caregiver encourages the child to shoplift (4)
**Emotional Maltreatment**	Emotional Abuse	Caregiver exposes child to continuous or extreme rejection or unpredictable/ threatening situations (commission)	Belittling the child (1)<br>Child expected to look after caregiver (2)<br>witnessing domestic violence (3/4)
	Emotional Neglect	Caregiver ignores/disregards child's basic emotional needs for safety, acceptance and autonomy (omission).	Child's need for comfort is ignored (2)<br>Caregiver abandons child and remains unresponsive to contact-attempts (4).

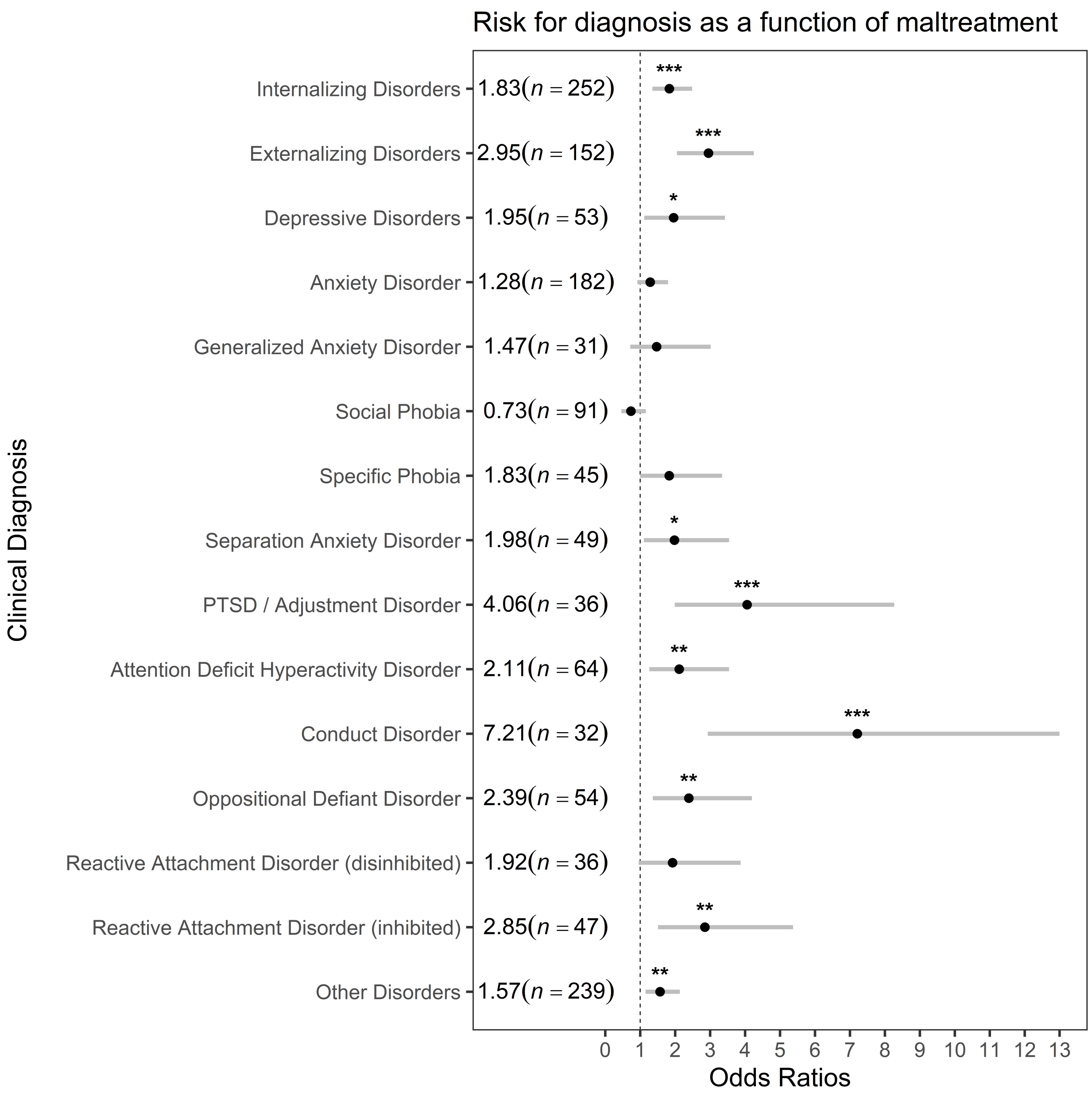

Figure S2. Odds ratios of developing any DSM, internalizing and/or externalizing disorders of maltreated (any subtype) compared to nonmaltreated youth (only diagnoses with caseload of $n \geq 30$ included).

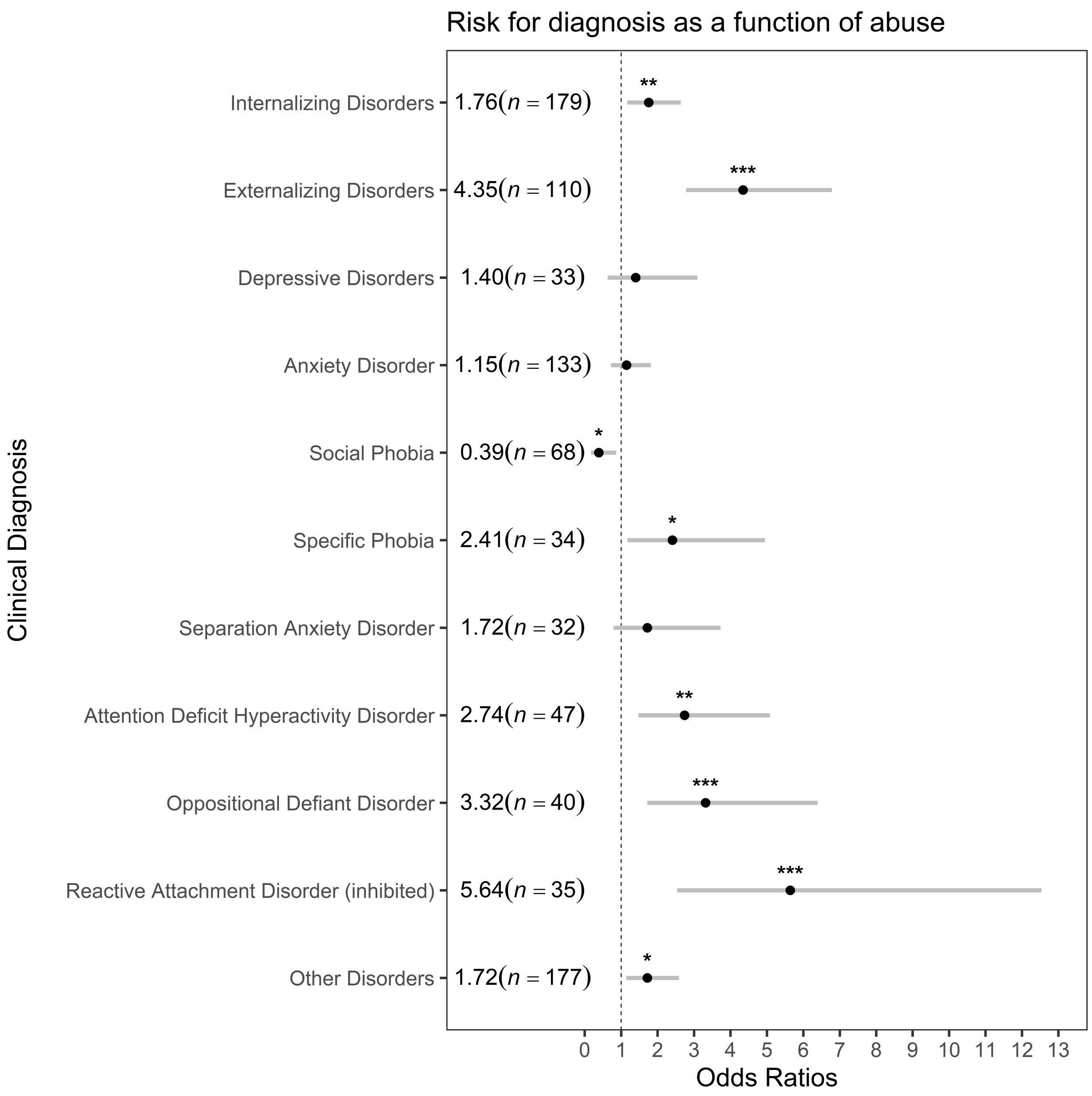

Figure S3. Odds ratios of developing any DSM, internalizing and/or externalizing disorders of abused compared to nonabused youth (only diagnoses with caseload of $n \geq 30$ included).

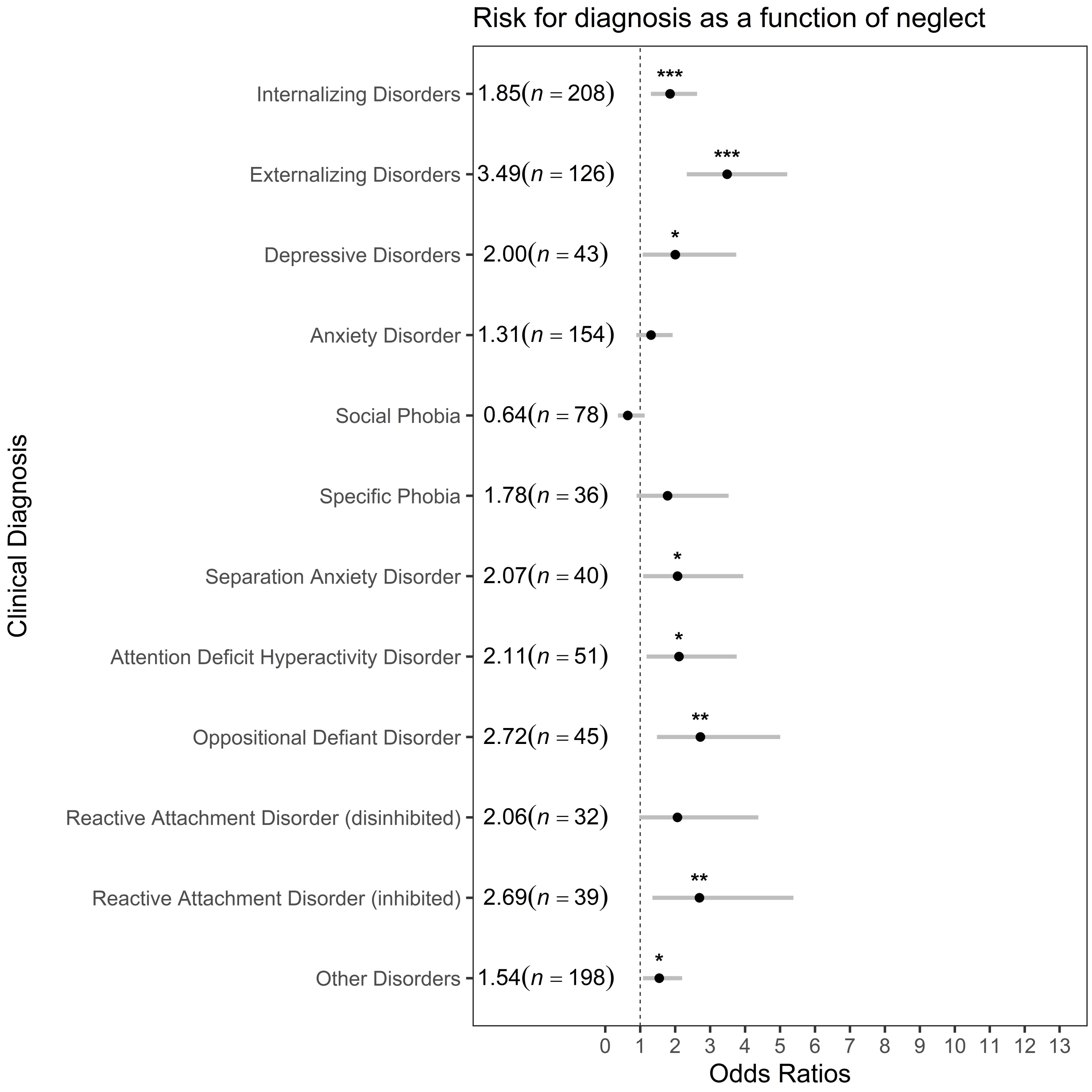

Figure S4. Odds ratios of developing any DSM, internalizing and/or externalizing disorders of neglected compared to nonneglected youth (only diagnoses with caseload of $n \geq 30$ included).

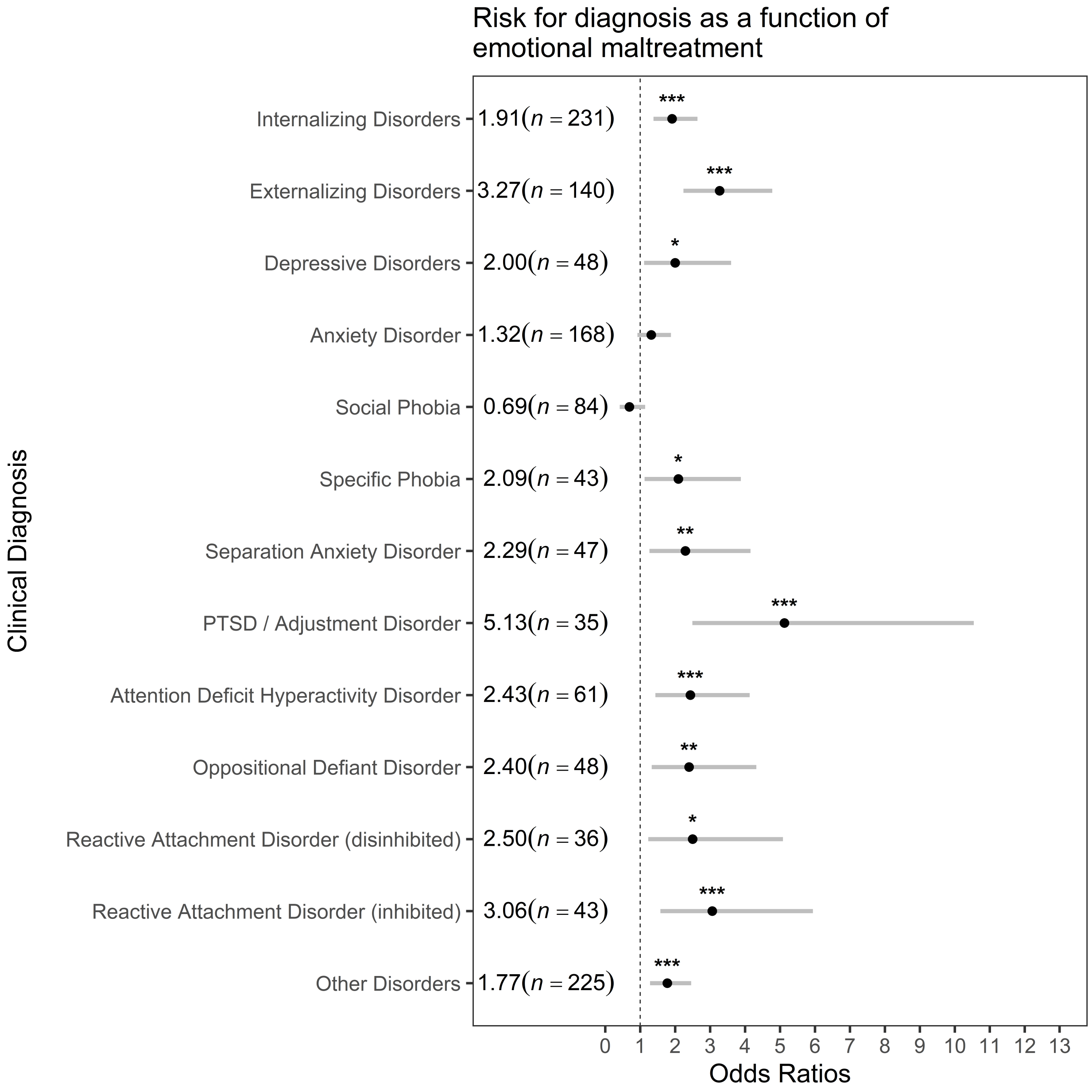

Figure S5. Odds ratios of developing any DSM, internalizing and/or externalizing disorders of emotionally maltreated compared to emotionally nonmaltreated youth (only diagnoses with caseload of $n \geq 30$ included).

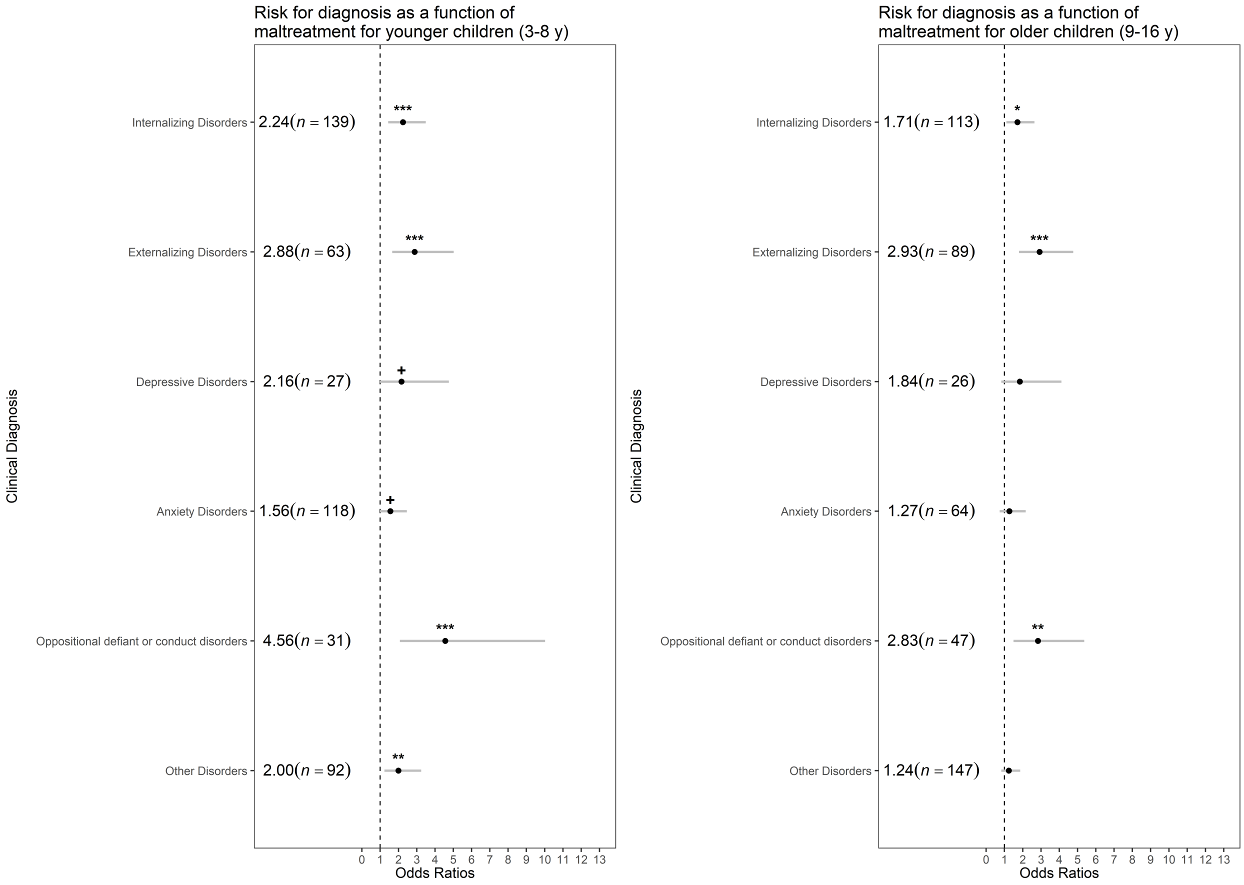

Figure S6. Odds ratios of developing internalizing, externalizing and/or other disorders of younger and older maltreated compared to nonmaltreated youth (only diagnoses with caseload of $n \geq 20$ included).

Table S3. Results of structural equation models investigating the effects of 1) maltreatment, 2) abuse and neglect (excl. emotional abuse and neglect), 3) abuse and neglect (incl. emotional abuse and neglect) and 4) emotional maltreatment on internalizing and externalizing disorders.

	**Model 1**	**Model 2**	**Model 3**	**Model 4**
	Stand.Coeff.(SE)	*Stand.Coeff.(SE)*	*Stand.Coeff.(SE)*	*Stand.Coeff.(SE)*
		**Factor Loadings**		
**INT**				
Depression	0.61*** (0.09)	0.56*** (0.09)	0.61*** (0.10)	0.59*** (0.10)
Anxiety	0.57*** (0.08)	0.51*** (0.08)	0.49*** (0.08)	0.45*** (0.08)
Other INT	0.75*** (0.11)	0.85*** (0.13)	0.84*** (0.13)	0.89*** (0.14)
**EXT**				
CD	0.87*** (0.09)	0.87*** (0.09)	0.90*** (0.10)	0.88*** (0.09)
ODD	0.57*** (0.08)	0.58*** (0.09)	0.55*** (0.09)	0.55*** (0.09)
ADHD + other EXT	0.58*** (0.07)	0.57*** (0.07)	0.57*** (0.08)	0.58*** (0.08)
**MAL**				
Nr. of Subtype	0.95*** (0.01)			
Chronicity	0.94*** (0.03)			
Severity	0.90*** (0.01)			
**ABU (excl. emotional abuse)**				
Nr. of Subtype		0.96*** (0.01)		0.95*** (0.01)
Chronicity		0.87*** (0.02)		0.87*** (0.04)
Severity		0.86*** (0.01)		0.87*** (0.02)
**NEG (excl. emotional neglect)**				
Nr. of Subtype		0.99*** (0.01)		0.99*** (0.01)
Chronicity		0.91*** (0.01)		0.89*** (0.03)
Severity		0.85*** (0.02)		0.87*** (0.02)
**ABU+ (incl. emotional abuse)**				
Nr. of Subtype			0.94*** (0.01)	0.95*** (0.01)
Chronicity			0.90*** (0.01)	0.87*** (0.04)
Severity			0.91*** (0.01)	0.87*** (0.02)
**NEG+ (incl. emotional neglect)**				
Nr. of Subtype			0.95*** (0.01)	0.99*** (0.01)
Chronicity			0.92*** (0.01)	0.89*** (0.03)
Severity			0.85*** (0.02)	0.87*** (0.02)
**EM**				
Nr. of Subtype				0.92*** (0.01)
Chronicity				0.87*** (0.02)
Severity				0.98*** (0.01)
**AGE**				
Age (manifest)	1.00^{+}	1.00^{+}	1.00^{+}	1.00^{+}
**SEX**				
Sex (manifest)	1.00^{+}	1.00^{+}	1.00^{+}	1.00^{+}
**EDU**				
Car. Edu.(manifest)	1.00^{+}	1.00^{+}	1.00^{+}	1.00^{+}
		**Regression Slopes**		
**INT**				
MAL	0.19** (0.07)			
ABU		0.19* (0.07)		0.11 (0.07)
NEG		−0.02 (0.07)		−0.12 (0.08)

(cont'd on following page)

	Model 1		Model 2		Model 3		Model 4	
	Stand.Coeff.(SE)		*Stand.Coeff.(SE)*		*Stand.Coeff.(SE)*		*Stand.Coeff.(SE)*	
ABU+					0.30***	(0.08)		
NEG+					−0.07	(0.08)		
EM							0.26**	(0.08)
AGE	−0.15**	(0.05)	−0.14*	(0.05)	−0.13*	(0.05)	−0.12*	(0.05)
SEX	0.00	(0.06)	−0.01	(0.06)	−0.00	(0.06)	0.00	(0.06)
EDU	−0.10	(0.06)	−0.13	(0.06)	−0.09	(0.06)	−0.08	(0.06)
**EXT**								
MAL	0.36***	(0.06)						
ABU			0.23***	(0.06)			0.17**	(0.07)
NEG			0.14*	(0.06)			0.06	(0.07)
ABU+					0.28***	(0.07)		
NEG+					0.10	(0.07)		
EM							0.19*	(0.08)
AGE	0.06	(0.07)	0.05	(0.07)	0.07	(0.07)	0.05	(0.07)
SEX	−0.27***	(0.07)	−0.28***	(0.07)	−0.27***	(0.07)	−0.28***	(0.07)
EDU	−0.13	(0.06)	−0.17*	(0.06)	−0.14*	(0.06)	−0.13*	(0.06)
**Fit Indices**								
χ^2(df)	1273.391(66)***		2683.380(105)***		2331.745(105)***		4297.371(153)***	
CFI	.932		.960		.955		.968	
RMSEA	.052		.045		.044		.043	
SRMR	.079		.067		.066		.058	

Note. $^{*}p<.05$, $^{**}p<.01$, $^{***}p\leq .001$; $^{+}$ Fixed parameter.

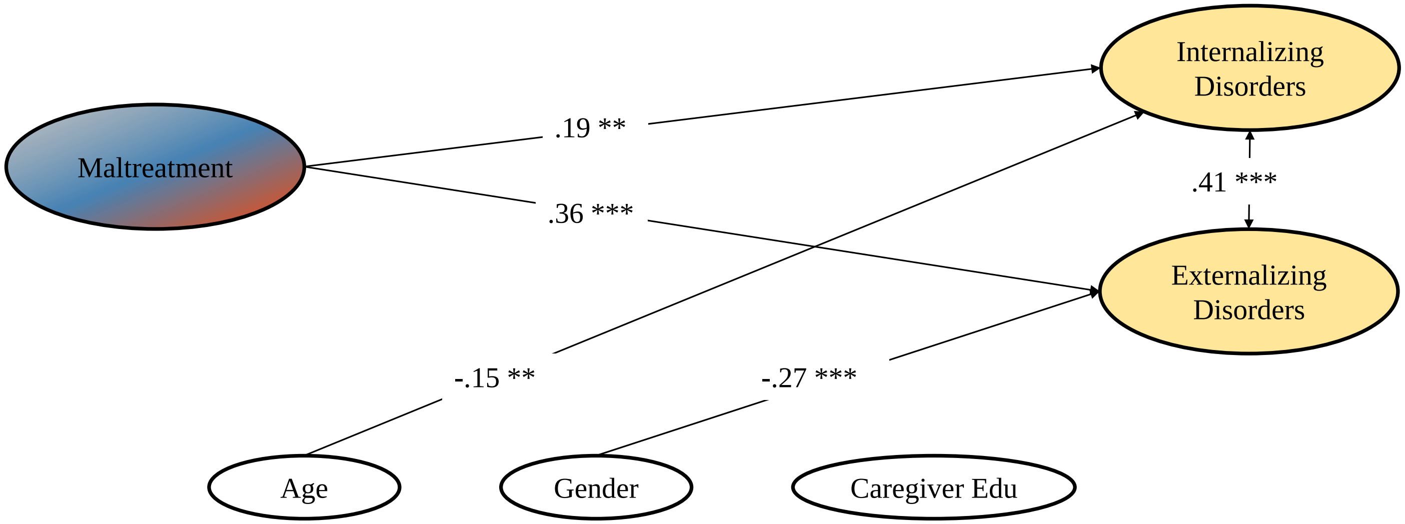

* *p*<.05; ** *p*<.01; *** *p*≤.001

Figure S7. Structural equation model 1 predicting psychiatric outcomes from maltreatment experiences after controlling for age of diagnosis, gender, and caregiver education.

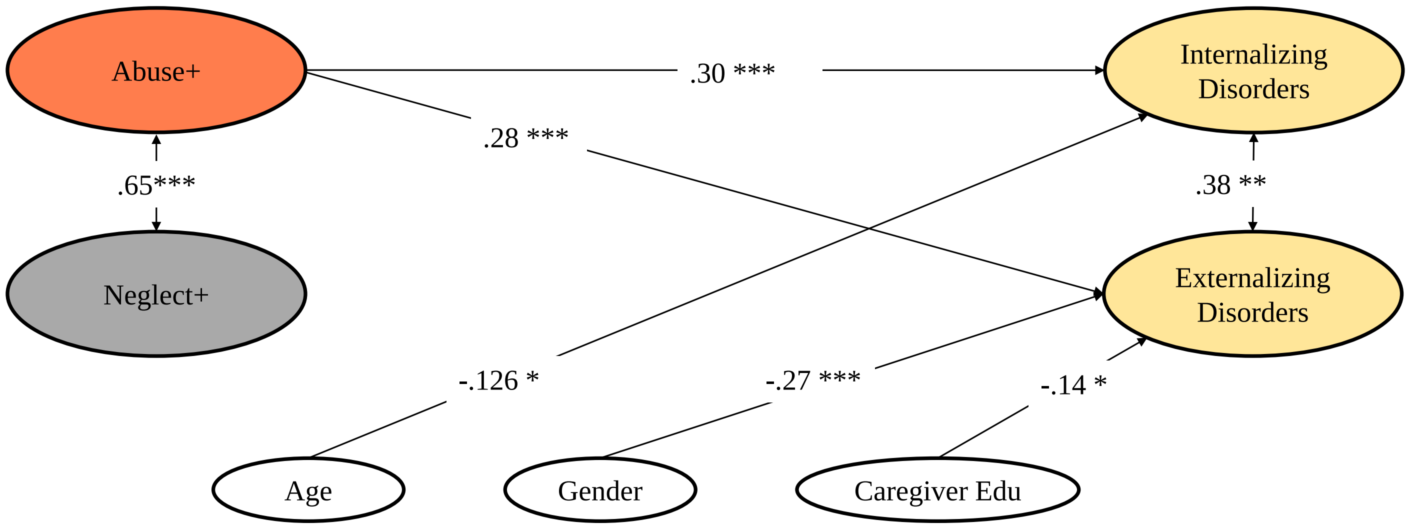

* p<.05; ** p<.01; *** p≤.001

Figure S8. Structural equation model 3 predicting psychiatric outcomes from abuse (including emotional abuse) and neglect (including emotional neglect) under the corresponding factors, after ontrolling for age of diagnosis, gender, and caregiver education.

Additional information on structural equation modelling (SEM)

Latent factors for maltreatment and diagnostic outcomes were modelled as follows:

1. The latent internalizing disorder factor was specified by (a) Sum of Depressive Disorders (Major Depression, Dysthymia, Depression – Not Other Specified, Bipolar Disorder), (b) Sum of Anxiety Disorders (Specific Phobia, Separation Anxiety Disorder, Generalized Anxiety Disorder, Social Phobia, Panic Disorder, Agoraphobia, Obsessive Compulsive Disorder, Selective Mutism), and (c) presence of any other disorder related to the internalizing spectrum (PTSD/ Adjustment Disorder, Inhibited Reactive Attachment Disorder, Eating and Tic Disorders).

2. The latent externalizing disorder factor was specified by (a) presence of Conduct disorder, (b) presence of Oppositional Defiant disorder, and (c) sum of presence of Attention Deficit/ Hyperactivity Disorder and presence of any other disorder related to the externalizing spectrum (Substance Use, Disinhibited Reactive Attachment Disorder, Encopresis).

3. The latent maltreatment dimensions (abuse, neglect and emotional maltreatment) were each specified by three indicators: (1) maximum severity of the subtype across development, (2) chronicity (i.e., the proportion of periods affected by a subtype relative to the total number of experienced periods), and (3) number of subtypes. As the MCS does not distinguish subtypes of emotional maltreatment, binary presence of emotional maltreatment was used as the third indicator for this dimension.

Table S4. Results of the structural equation model investigating the effects of abuse and neglect (excl. emotional abuse and neglect) and emotional maltreatment on internalizing and externalizing disorders, controlling for *witnessing domestic violence*.

	Model 4b	
	Stand.Coeff.(SE)	
	**Factor Loadings**	
**INT**		
Depression	0.57***	(0.10)
Anxiety	0.49***	(0.08)
Other INT	0.86***	(0.13)
**EXT**		
CD	0.85***	(0.09)
ODD	0.55***	(0.09)
ADHD + other EXT	0.61***	(0.07)
**ABU (excl. emotional abuse)**		
Nr. of Subtype	0.95***	(0.01)
Chronicity	0.88***	(0.01)
Severity	0.88***	(0.01)
**NEG (excl. emotional neglect)**		
Nr. of Subtype	0.99***	(0.01)
Chronicity	0.90***	(0.01)
Severity	0.86***	(0.02)
**EM without WDV**		
Nr. of Subtype	0.93***	(0.01)
Chronicity	0.80***	(0.02)
Severity	0.99***	(0.02)
**AGE**		
Age (manifest)	1.00^{+}	
**SEX**		
Sex (manifest)	1.00^{+}	
**EDU**		
Car. Edu.(manifest)	1.00^{+}	
**WDV (yes/no)**		
Car. Edu.(manifest)	1.00^{+}	
	**Regression Slopes**	
**INT**		
ABU	0.18**	(0.07)
NEG	−0.08	(0.08)
EM	0.16*	(0.07)
AGE	−0.14**	(0.05)
SEX	−0.00	(0.06)
EDU	−0.11	(0.06)
**EXT**		
ABU	0.27***	(0.06)
NEG	0.11	(0.07)
EM	0.03	(0.08)

(cont'd on following page)

	Model 4b	
	Stand.Coeff.(SE)	
AGE	0.07	(0.07)
SEX	−0.27***	(0.07)
EDU	−0.13*	(0.06)
	**Fit Indices**	
χ^2(df)	4615.654(153)***	
CFI	.966	
RMSEA	.046	
SRMR	.060	

Note. $^{*}p$ <.05, $^{**}p$ <.01, $^{***}p \leq$ .001; $^{+}$ Fixed parameter.

Table S5. Results of the post-hoc multigroup structural equation model investigating the effects of abuse and neglect (excl. emotional abuse and neglect) and emotional maltreatment on internalizing and externalizing disorders for younger (3-8) and older (9-16) youth.

	**Younger youth**		**Older youth**	
	Stand.Coeff.(SE)		*Stand.Coeff.(SE)*	
	**Factor Loadings**			
**INT**				
Depression	0.71***	(0.11)	0.41***	(0.15)
Anxiety	0.52***	(0.10)	0.51***	(0.12)
Other INT	0.96***	(0.15)	0.81***	(0.17)
**EXT**				
CD	0.95***	(0.11)	0.86***	(0.12)
ODD	0.66***	(0.12)	0.58***	(0.10)
ADHD + other EXT	0.03***	(0.14)	0.77***	(0.08)
**ABU (excl. emotional abuse)**				
Nr. of Subtype	0.97***	(0.02)	0.94***	(0.01)
Chronicity	0.92***	(0.02)	0.86***	(0.01)
Severity	0.91***	(0.02)	0.85***	(0.02)
**NEG (excl. emotional neglect)**				
Nr. of Subtype	0.97***	(0.02)	0.98***	(0.01)
Chronicity	0.93***	(0.02)	0.90***	(0.01)
Severity	0.90***	(0.02)	0.83***	(0.02)
**EM**				
Nr. of Subtype	0.95***	(0.01)	0.91***	(0.01)
Chronicity	0.91***	(0.03)	0.86***	(0.02)
Severity	0.98***	(0.02)	0.99***	(0.01)
**SEX**				
Sex (manifest)	1.00^{+}		1.00^{+}	
**EDU**				
Car. Edu.(manifest)	1.00^{+}		1.00^{+}	
	**Regression Slopes**			
**INT**				
ABU	0.16*	(0.07)	0.01	(0.10)
NEG	−0.09	(0.14)	−0.08	(0.10)
EM	0.19	(0.12)	0.29**	(0.10)
SEX	0.07	(0.08)	−0.07	(0.08)
EDU	0.01	(0.08)	−0.18	(0.09)
**EXT**				
ABU	0.03	(0.10)	0.30***	(0.07)
NEG	0.11	(0.09)	−0.01	(0.09)
EM	0.27*	(0.12)	0.09	(0.10)
SEX	0.04	(0.11)	−0.44***	(0.08)
EDU	−0.26**	(0.09)	−0.08	(0.08)
	**Fit Indices**			
χ^2(df)	4063.524(272)***			
CFI	.978			
RMSEA	.035			
SRMR	.071			

Note. $^{*}p < .05$, $^{**}p < .01$, $^{***}p \leq .001$; $^{+}$ Fixed parameter.
